# Hospital Pharmacists and Antimicrobial Stewardship: A Qualitative Analysis

**DOI:** 10.3390/antibiotics10121441

**Published:** 2021-11-24

**Authors:** Lok Hang Wong, Evonne Tay, Shi Thong Heng, Huiling Guo, Andrea Lay Hoon Kwa, Tat Ming Ng, Shimin Jasmine Chung, Jyoti Somani, David Chien Boon Lye, Angela Chow

**Affiliations:** 1 Department of Clinical Epidemiology, Office of Clinical Epidemiology, Analytics, and Knowledge, Tan Tock Seng Hospital, Singapore 308433, Singapore; lokhangj6@hotmail.com (L.H.W.); evonne_tay@ncid.sg (E.T.); huiling_guo@ttsh.com.sg (H.G.); 2 Infectious Disease Research and Training Office, National Centre for Infectious Diseases, Singapore 308443, Singapore; david_lye@ncid.sg; 3 Department of Pharmacy, Tan Tock Seng Hospital, Singapore 308433, Singapore; shi_thong_heng@ttsh.com.sg (S.T.H.); tat_ming_ng@ttsh.com.sg (T.M.N.); 4 Department of Pharmacy, Singapore General Hospital, Singapore 169608, Singapore; andrea.kwa.l.h@sgh.com.sg; 5 Programme in Emerging Infectious Diseases, Duke-NUS Medical School, Singapore 169857, Singapore; 6 Department of Infectious Diseases, Singapore General Hospital, Singapore 169608, Singapore; jasmine.chung.s.m@singhealth.com.sg; 7 Yong Loo Lin School of Medicine, National University of Singapore, Singapore 119228, Singapore; 8 Division of Infectious Diseases, National University Hospital, Singapore 119074, Singapore; somani_jyoti@nuhs.edu.sg; 9 Department of Infectious Diseases, Tan Tock Seng Hospital, Singapore 308433, Singapore; 10 Lee Kong Chian School of Medicine, Nanyang Technological University, Singapore 636921, Singapore

**Keywords:** antimicrobial stewardship, antimicrobial resistance, hospitals, hospital pharmacists, challenges, antibiotic prescribing

## Abstract

Antimicrobial stewardship programmes (ASPs) in hospitals are predominantly led by specific ASP physicians and pharmacists. Limited studies have been conducted to appreciate non-ASP-trained hospital pharmacists’ perspectives on their roles in antimicrobial stewardship. Focus group discussions (FGDs) were conducted with 74 pharmacists, purposively sampled from the 3 largest acute-care public hospitals in Singapore, to explore facilitators and barriers faced by them in antimicrobial stewardship. Applied thematic analysis was conducted and codes were categorised using the social–ecological model (SEM). At the intrapersonal level, pharmacists identified themselves as reviewers for drug safety before dispensing, confining to a restricted advisory role due to lack of clinical knowledge, experience, and empowerment to contribute actively to physicians’ prescribing decisions. At the interpersonal level, pharmacists expressed difficulties conveying their opinions and recommendations on antibiotic therapy to physicians despite frequent communications, but they assumed critical roles as educators for patients and their caregivers on proper antibiotic use. At the organisational level, in-house antibiotic guidelines supported pharmacists’ antibiotic interventions and recommendations. At the community level, pharmacists were motivated to improve low public awareness and knowledge on antibiotic use and antimicrobial resistance. These findings provide important insights into the gaps to be addressed in order to harness the untapped potential of hospital pharmacists and fully engage them in antimicrobial stewardship.

## 1. Introduction

Antimicrobial resistance (AMR) is a rising public health threat with new resistant bacteria emerging more rapidly than novel antibiotics being developed, threatening the effectiveness of existing antibiotic treatment options [1,2]. Studies show that 30% to 50% of prescribed antibiotics in hospitals were either unnecessary or inappropriate [3], a factor which is a major driver of AMR. AMR can complicate the treatment of simple infections, prolong the length of hospital stay, and increase treatment costs and mortality rates [1,4]. To monitor and promote appropriate antibiotic use in hospitals, antimicrobial stewardship programmes (ASPs) were introduced in all public acute-care hospitals in Singapore since 2011 [5,6].

ASPs have increased appropriate antibiotic prescribing and reduced the length of hospital stay without compromising patient safety, providing valuable clinical and economic benefits [7,8]. However, antibiotic use remains high in many developed countries, with one in two hospitalised patients prescribed at least one antimicrobial agent per day [9,10].

Pharmacists are well versed in the pharmacology of antibiotics and are expected to review prescriptions and advise physicians on the most appropriate antibiotic choice and regimen [11]. In its 2019 guidelines, US Centres for Disease Control and Prevention emphasised the importance of pharmacy expertise, by promoting ‘handshake stewardship’ between physicians and pharmacists for more effective implementation of ASPs in hospitals [12].

Hospital pharmacists have traditionally assumed responsibilities in the governance of medications such as prior authorisation for restricted drugs and provision of audit and feedback to prescribing physicians. However, their perceived role in antimicrobial stewardship has been predominantly advisory in nature and focused on the assurance of drug safety [13]. The role of pharmacists has appeared to be limited by pharmacists’ perception of medico-legal responsibilities and the prescribing authority vested in physicians [14]. Their role in antimicrobial stewardship is further limited by the lack of training and specialisation in infections, antibiotic spectrum, and antimicrobial stewardship [15], prevailing hospital culture, and resource constraints (such as the inadequacy of electronic surveillance systems). Hospital pharmacists need to be equipped with the necessary knowledge, skills, and tools to play an enhanced role in optimising antibiotic use [16].

Whilst many established hospital ASPs include infectious disease (ID) pharmacists, these dedicated ASP pharmacists are by far few in number. Other hospital pharmacists remain an untapped resource for antimicrobial stewardship activities. This study aims to identify facilitators and barriers to non-ASP hospital pharmacists’ participation in antimicrobial stewardship and explore challenges faced in influencing optimal antibiotic use in the three largest acute-care hospitals in Singapore. An in-depth understanding of these factors can influence the development and implementation of interventions to empower hospital pharmacists to play more significant roles in antimicrobial stewardship.

## 2. Material and Methods

### 2.1. Study Design and Study Population

Focus group discussions (FGDs) were conducted with non-ASP hospital pharmacists purposively sampled from 1200-bed National University Hospital (NUH), 1700-bed Tan Tock Seng Hospital (TTSH), and 1785-bed Singapore General Hospital (SGH) in Singapore between November 2018 and April 2019.

In each of the hospitals, there are 5–10 ASP and 70–80 non-ASP inpatient hospital pharmacists. Non-ASP hospital pharmacists are responsible for dispensing, counselling, supply, and evaluation of medication use. In general, non-ASP pharmacists review the appropriateness of medication use pertaining to the indications, dosing, and administration, and intervene on inappropriate orders. Additionally, the more experienced non-ASP pharmacists join ward rounds with physicians and optimise pharmacotherapy for patients [17]. Since 2011, the three hospitals have established ASP teams which include 5–10 full-time ASP pharmacists dedicated to the active review and feedback to prescribing physicians on the inappropriate use of broad-spectrum antibiotics, development, and updating of antimicrobial use guidelines, and education on antimicrobial stewardship [18].

Non-ASP hospital pharmacists were invited to participate in the FGDs via invitation letters sent through the pharmacy department in each hospital. Interested pharmacists contacted the study team, who would then purposively select eligible participants into the study to ensure good representation with maximum variation. Participants were subsequently grouped into the respective FGDs based on their seniority and institution to minimise social desirability bias. This was also to ensure that participants were able to freely share their views and opinions during the FGDs.

To achieve maximum variation, four FGDs of 4–8 hospital pharmacists who have practised for at least one year in their respective institutions were conducted in each hospital; for each institution, 1–2 FGDs of junior pharmacists, 1–2 of senior pharmacists, and 1 of principal/clinical/specialist pharmacists were conducted. Junior pharmacists were defined as pharmacists with ≤4 years of hospital experience, while senior pharmacists were pharmacists with >4 years of experience. Principal/clinical/specialist pharmacists were pharmacists with specialised training in specific clinical specialties (but not in ASP) who had ≥8 years of experience and spent >50% of their time in direct patient care.

### 2.2. Data Collection

Informed consent was taken prior to the commencement of each FGD. Two Research Assistants (both females and with a Bachelor’s degree of a non-healthcare major) trained in qualitative data collection techniques were present as a facilitator and a note-taker during each FGD session. The FGDs were conducted in a closed-door meeting room within the respective hospital premises. To protect their identities, study participants chose their own pseudonyms and were addressed by those chosen pseudonyms during the FGDs. FGDs were audio recorded and lasted approximately 1.5–2 h.

A semi-structured interview guide was developed based on existing literature and piloted with ASP pharmacists who understood the roles of non-ASP pharmacists to ensure the topic guide was comprehensive [13,19,20]. The interview guide was designed to explore the roles and involvement of hospital pharmacists in antimicrobial stewardship, their views and perceptions about the use of antibiotics in their respective institutions, as well as their awareness and attitudes towards AMR (refer to the Topic Guide in Supplementary File (Annex S1) for further details). Basic demographic data were collected from each participant. The study was conducted and reported according to the Consolidated Criteria for Reporting Qualitative Research (COREQ) guidelines [21] (Supplementary File (Annex S2)).

### 2.3. Data Analysis

All FGDs were transcribed verbatim and reviewed for accuracy by a third study team member. A preliminary codebook was subsequently developed using the interview guide questions and after data familiarisation with the first few transcripts. Three coders coded a randomly selected transcript, and inter-coder reliability was then ascertained by comparing and resolving any discrepancies in the coding process. Data saturation was also discussed. The process was repeated to arrive at the final codebook which was used to code the remaining transcripts. Codes were organised according to the social–ecological model (SEM) [22], using QSR International’s NVivo 12 software, then summarised and analysed using applied thematic analysis [23]. SEM emphasises the interaction between multiple levels of influence at the intrapersonal, interpersonal, social, and organisational levels. Identification of these factors could effectively strategise interventions to enhance the role of non-ASP hospital pharmacists in antimicrobial stewardship. Themes were documented from the perspective of the pharmacists on their ability to intervene on antibiotic choices, promote therapeutic guidelines and interact with physicians, as well as on any constraints limiting their participation in antibiotic prescribing decisions.

## 3. Results

In total, 12 FGDs comprising of 74 non-ASP inpatient hospital pharmacists were conducted (Table 1). All participants had at least a Bachelor’s degree, with one-quarter of them having had postgraduate education (Masters or Doctorate). The years of working experience in their respective hospitals ranged from 2 to 24 years, with a median of 5 years.

**Figure 1:**
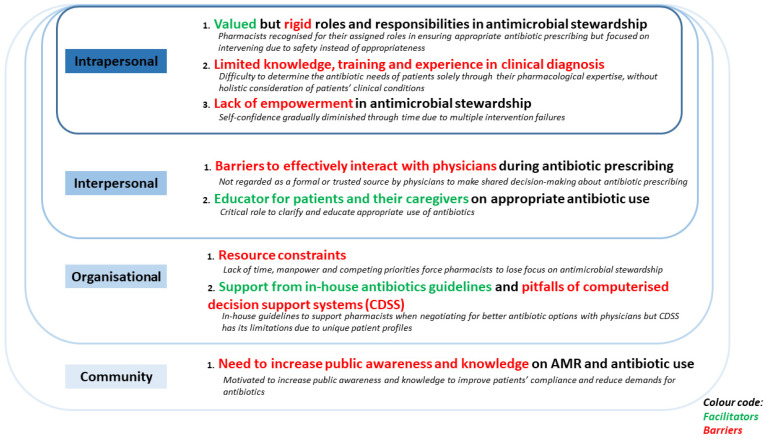
Social-ecological model (SEM) elucidating the facilitators and barriers faced by non-ASP hospital pharmacists during antimicrobial stewardship (McLeroy et al., 1988).

Facilitators and barriers faced by hospital pharmacists during antimicrobial stewardship were categorised into the intrapersonal, interpersonal, organisational, and community levels, and summarised in Figure 1.

### 3.1. Intrapersonal Level

At the intrapersonal level, pharmacists described their roles and responsibilities in antimicrobial stewardship. They emphasised their priorities and shared their clinical experiences and knowledge of antibiotic prescribing, as well as their empowerment to intervene on antibiotic prescribing decisions by physicians.

#### 3.1.1. Valued but Rigid Roles and Responsibilities in Antimicrobial Stewardship

Most pharmacists described their roles in the review of antibiotic orders, and their responsibilities in ensuring the correct dosage and appropriateness of antibiotics. At times, they would suggest the de-escalation of antibiotic therapy. Senior pharmacists mentioned being more involved in discussions with the medical team during antibiotic prescribing, as compared with junior pharmacists.

*“So when we review the antibiotics, we will check the appropriateness and the duration and all sorts, and when is the best time to oralise or maybe de-escalate the antibiotic. So it depends on the situation, if it’s empiric then we would check whether is it appropriate … we would check the culture to see whether is there any need to change to a more targeted therapy.”*

(FGD010, Senior pharmacist)

*“I think sometimes if we round with the physicians then we will discuss upon initiation. What antibiotics to start and also… and later on what antibiotics to de-escalate to.”*

(FGD003, Senior pharmacist)

*“As long as the dose is safe for the patient, and the duration is not outrightly wrong, we will generally, let it go.”*

(FGD001, Junior pharmacist)

When intervening on an antibiotic order, most pharmacists prioritised safety over the appropriateness of the antibiotic. When antibiotics prescribed by physicians were not the most appropriate, as long as the prescriptions were within safety limits, pharmacists would allow the antibiotics to be dispensed.

*“… I think we would [only] really do something if safety… is an issue. We just make sure everything is safe and appropriate, then I think it’s okay.”*

(FGD005, Junior pharmacist)

*“Oh I mean certain consultants, they have their own preferred combination of antibiotics [sometimes]… So as long as it is not going to cause major harm… Then we may still go ahead with the antibiotics.”*

(FGD008, Principal pharmacist)

#### 3.1.2. Limited Knowledge, Training, and Experience in Clinical Diagnosis

Whilst the majority of pharmacists were confident in their pharmacology knowledge, they often felt incompetent to refute the medical team’s decision. Without knowledge and training in clinical diagnosis, pharmacists felt that it was difficult to determine the antibiotic needs of patients based solely on microbiological results without holistic consideration of patients’ clinical conditions.

*“From a pharmacist point of view I think we can dose better, I mean our knowledge now is still restricted to dosing, I think with regards to wound examination everything I don’t think we know anything much also.”*

(FGD005, Junior pharmacist)

*“So because it has to do with diagnostics, when it has to do with [something] like diagnostics. We, we don’t have a say. We can’t review the chest X-ray and just say the patient has no pneumonia, because we are not clinically trained to do that.”*

(FGD003, Senior pharmacist)

When in doubt, junior pharmacists would consult their seniors, and if discrepancies arose, they would escalate to ASP pharmacists.

*“If we [are] still stuck then we will check with our colleagues whoever that has more experience in that area, whether they [have] seen this before and if still unable to [resolve], then maybe we will ask those seniors [in] ASP or ICU pharmacist.”*

(FGD010, Senior pharmacist)

#### 3.1.3. Lack of Empowerment in Antimicrobial Stewardship

Some pharmacists felt that it was necessary to be confident of their recommendations after thorough checks, before suggesting an intervention to the medical team, especially since they had not clinically attended to the patients.

*“To make recommendations, [it depends on] how complex the case is. So if [it] is so complex, I don’t want [to make] any recommendations, I’m not confident also. Sometimes, for us it’s also a bit of a snapshot.”*

(FGD003, Senior pharmacist)

Moreover, pharmacists lacked confidence that their interventions would improve patient outcomes and expressed concerns over medical liabilities.

*“If they really heed our advice and patient deteriorates… then whose responsibility [would it be]. This is a very big concern actually.”*

(FGD003, Senior pharmacist)

In many FGDs, junior pharmacists mentioned that they had intervened actively on antibiotic orders at the start of their careers. However, due to multiple failed attempts at changing physicians’ prescribing habits, they had reduced interventions on the appropriateness of antibiotics and instead, focused on ensuring drug safety.

*“So if we know that this particular team is always doing this thing, then in the future if I see the same thing happen, I won’t… I will know that… I may still try but won’t try so hard.”*

(FGD010, Senior pharmacist)

*“Yes, in a way, because there are some consultants that you know that no matter what you say they are not going to change their mind. Then you don’t bother saying anymore.”*

(FGD012, Principal pharmacist)

As such, many pharmacists shared that their contributions towards antimicrobial stewardship were very limited.

*“There…there’s very limited, things we can do. Because… Firstly, we’re not able to prescribe and secondly, uh, they [physicians] can use reasons like “I think the patient is clinically unwell”. Yes. So…there’s no way that you could actually… argue back* *.”*

(FGD001, Junior pharmacist)

### 3.2. Interpersonal Level

At the interpersonal level, pharmacists shared their experiences and perceptions of working with physicians during antibiotic prescribing and educating patients and caregivers.

#### 3.2.1. Barriers to Effectively Interact with Physicians during Antibiotic Prescribing

Across all institutions, pharmacists shared that they were commonly consulted by physicians on antibiotic dosing and frequency for patients with impaired renal function, dosing suggestions for antibiotics that require therapeutic drug monitoring, and antibiotic choices for patients with multiple drug allergies.

*“It’s culture directed, they don’t know what to choose, or [only] if the person is penicillin allergic, or a lot of allergies, only then they will ask you. Most of the time, if it’s a clear cut situation, then they will make a decision on their own.”*

(FGD006, Senior pharmacist)

Frequently, pharmacists felt that their concerns about inappropriate use of antibiotics could not be explicitly communicated to prescribing physicians, and their suggestions were often ignored, as they were not formally part of the clinical decision-making process for antibiotic prescribing.

*“I do think sometimes there’s a psychological barrier to communicate with the team… I guess there’s a need for us to actually really speak up… [like] our idea[s] and our concerns and communicate not just with HO [House Officer], MO [Medical Officer], [but] try to move up and because sometimes we are afraid to actually voice out our concern”*

(FGD005, Junior pharmacist)

*“Like for example the surgical disciplines, they tend to start antibiotics without any strong indication. Or they will just put an IDC [indwelling catheter] and then they will start ciprofloxacin, sometimes at the wrong doses. But [if] you ask them to off, [they will reply] no, it’s the consultant’s decision.”*

(FGD002, Junior pharmacist)

Some pharmacists emphasised that it was particularly difficult to intervene on antibiotic choices for senior physicians, as they might favour the use of certain antibiotics based on their past clinical experiences and/or personal preferences. Most of their recommendations were usually overridden by the decision of the senior physician in the primary medical team.

*“I mean I guess there [are] always certain teams or physicians who probably have their own rationale or mindset in terms of like why would they still prefer to use certain choices. I mean they probably have certain experience in the past.”*

(FGD007, Junior pharmacist)

*“…so when we do the interventions, or we can, we talk to the junior doctors about that, uh they will say follow the consultants, so they will follow the consultants past experience…”*

(FGD011, Principal pharmacist)

This was despite the fact that physicians might not have the best knowledge on the appropriate use of antibiotics.

*“I think one very common one is [that] younger doctors might not know the concept of ESBL [extended-spectrum beta-lactamase] like some bacteria that is resistant to. So the panel will reflect this sensitive to Augmentin bu-, but we’re usually taught that the preferred one is the carbapenems. Then the doctor will question and say “It’s sensitive so why shouldn’t I use it?”*

(FGD002, Junior Pharmacist)

However, the pharmacists also mentioned that barriers to effective interventions could be mitigated by joining the medical rounds with physicians and participating in discussions at the initiation of antibiotics.

*“Usually we review them when the doctor orders the antibiotic. In terms of indication, the drug doses. And, in doubt, we clarify with the team regarding the use. Sometimes if we round with the physicians, then we will discuss upon initiation.”*

(FGD003, Senior pharmacist)

Overwhelmingly, hospital pharmacists felt that physicians tended to accept the antibiotic recommendations from ASP pharmacists or infectious disease physicians more than theirs, despite them providing the same recommendations.

*“Even if it’s just for prophylaxis…I do not know a lot about the surgical stuff, I do not have a very good solid reason to tell them “hey you must stop now…” And partly what we say does not carry the same weight as what the ASP [says].”*

(FGD004, Junior pharmacist)

*“I mean if the intervention comes from a ID consultant. Then all the more [it] would [be] accept[ed] compared to [the intervention] coming from [a] pharmacist right?”*

(FGD012, Principal pharmacist)

*“So what I recommended wasn’t wrong, It’s just that it needs to come out from a person that [is] more trustable.”*

(FGD004, Junior pharmacist)

#### 3.2.2. Educator for Patients and Their Caregivers on Appropriate Antibiotic Use

Most pharmacists interacted with patients and their caregivers when preparing patients for discharge. The pharmacist would educate patients or their caregivers on the purpose of the antibiotics and their proper use. They played the role of educators by clarifying patients’ and caregivers’ doubts and misconceptions and would emphasise the need to complete the entire course of antibiotics. Pharmacists expressed that patients and caregivers were usually trusting and accepting of the medical team’s decision to prescribe the antibiotics, as well as the pharmacists’ explanation.

*“So, we just tell them the general condition may have a lot of different [coughing] kinds of bugs. So, each antibiotic targets a different kind, so that’s why they need…more than one type. But they will generally accept the…our justification and counselling.”*

(FGD001, Junior pharmacist)

*“[During] dispensing, it will usually be the oral antibiotic, so just telling them how to use it. Or sometimes they will ask you simple questions like whether this antibiotic is considered strong, will it have a lot side effects, so it’s just to kind of educate them on what is it, and why is it important for you to take [that] you need to finish the course.”*

(FGD006, Senior pharmacist)

### 3.3. Organisational Level

At the organisational level, pharmacists discussed various facilitators and barriers to their contributions to antimicrobial stewardship in their daily clinical work.

#### 3.3.1. Resource Constraints

The lack of manpower, time constraints, and competing priorities were the main barriers to antimicrobial stewardship. With a heavy workload in a fast-paced working environment, non-ASP pharmacists were less likely to negotiate with physicians on the appropriate antibiotic therapy.

*“Sometimes it’s just in our culture because everything is so fast and you know [name of institution] is very busy. So we don’t have the time to just stop and listen [to the physicians’ rationales].”*

(FGD005, Junior pharmacist)

Furthermore, as antimicrobial stewardship was not a key performance indicator for non-ASP pharmacists, it was low in priority.

*“For ID [Infectious Diseases department],* *of course that would be their KPIs [Key Performance Indicators]. But let’s say if you have a lot of things on mind, … your main job is just to, like for pharmacist, would just be dispensing, reviewing orders. How much details can you go into? Dwelling into drug resistance that would be my last line.”*

(FGD011, Principal pharmacist)

#### 3.3.2. Support from In-House Antibiotic Guidelines and Pitfalls of Computerised Decision Support Systems

In-house antibiotic guidelines were perceived by most participants as being useful in supporting their interventions and antibiotic recommendations. Some pharmacists felt that physicians tended to be more receptive when their recommendations were based on guidelines, as they were evidence-based and approved by infectious disease physicians.

*“Easier to back up actually. Like you can tell them [physicians] as for [name of hospital] guidelines recommend to use this antibiotics, if you have this, this, this… They [are] a bit more receptive cause it’s guided by our institution.”*

(FGD006, Senior pharmacist)

However, physicians’ compliance with the hospital guidelines varied between clinical departments.

*“There is varying compliance with the guidelines. Also because the different departments deal with very different kind of patients. So that, the practice is still quite mixed.”*

(FGD012, Principal pharmacist)

Antibiotic computerised decision support systems (CDSSs) were available in two study institutions (TTSH and SGH). In general, hospital pharmacists who had the experience of using an antibiotic CDSS felt that whilst CDSS could guide antibiotic prescribing, the algorithms were non-exhaustive and might not address all patient situations, such as patients with multiple antibiotic allergies. Furthermore, one participant shared that human judgment was still required for antibiotic prescribing decisions.

*“Even though the ARUS-C [referring to TTSH’s institution-specific CDSS, the Antimicrobial Resistance Utilisation and Surveillance Control system] says or whatever guideline ARUS-C sets, it’s not like we have to follow it 100% so… I mean it’s useful for juniors or someone who’s not familiar. But if [for] someone that was experienced, it’s good to rely on the critical thinking rather than just follow the guidelines.”*

(FGD011, Principal pharmacist)

### 3.4. Community Level

At the community level, hospital pharmacists perceived that the general public had poor awareness of AMR and knowledge on antibiotic use.

#### Need to Increase Public Awareness and Knowledge on AMR and Antibiotic Use

Pharmacists mentioned the need to instil public awareness of AMR, which might help improve patients’ compliance with antibiotics. Educating and enhancing the public’s understanding of AMR and the use of antibiotics would reduce the pressure on the medical team to prescribe antibiotics due to patient demands.

*“I think layman education… very important. If there is [knowledge] there is less, less request then less pressure to prescribe.”*

(FGD003, Senior pharmacist)

*“[For] patients, will be [public] education, the more they know about their condition, the more they understand, and maybe they won’t feel that I must have antibiotics.”*

(FGD006, Senior pharmacist)

Pharmacists cited instances when patients expected antibiotics even though their medical conditions did not require them, and how their demands resulted in unnecessary antibiotics prescribed. Moreover, hospital pharmacists were concerned about patients’ compliance with antibiotics due to their lack of awareness and threat of AMR.

*“I mean we have patients who come in for minor surgery and … they expect antibiotic and they make a fuss at the counter and ask for antibiotic and because there’s a fuss, I have to get the doctor, okay can you just give some antibiotic.”*

(FGD004, Junior pharmacist)

*“So some of them, I have patients before who ask for standby antibiotics on discharge to bring home, because they, and I don’t know whether they know how to use it appropriately but sometimes the team, they cannot turn the patient down also.”*

(FGD005, Junior pharmacist)

### 3.5. Suggestions to Improve the Role of Non-ASP Hospital Pharmacists in Antimicrobial Stewardship

Despite the challenges faced, non-ASP hospital pharmacists continued to recognise the important role they play in antimicrobial stewardship and had suggested leveraging their ongoing roles in medication reviews to opportunistically ensure the optimisation of antibiotics.

*“I think we do play a role in optimising the dosage of the antibiotic. I know we don’t get to tell them “you know you shouldn’t use the antibiotic”, but once the antibiotic is started, we can be the one that optimise the dose to make sure that when we are giving a therapeutic dose, treat it well, [not] to develop resistance, in that sense. [So yes], I think we can play a part in educating the doctors so when we call to intervene [we can make them aware].”*

(FGD004, Junior Pharmacist)

Furthermore, the pharmacists advocated the use of institutional guidelines to facilitate their antimicrobial stewardship efforts.

*“[With] institutional guidelines in place. It is easier for people to follow. Especially good educational tools for junior doctors on the ground, also easier for pharmacists to use it as a back-up when they are discussing with the doctors.”*

(FGD009, Senior pharmacist)

Finally, non-ASP pharmacists suggested more on-the-job training to better equip them to enhance their contributions to antimicrobial stewardship.

*“But what we can do as of now I think is quite limited… You [will] need probably more training as well to know. When you can de-escalate because sometimes you are not [as] clinically, that well trained like doctors. We don’t have [the] physical exam skills, we don’t know how to see if the patient is toxic, maybe our choice or our decision to escalate or de-escalate may not be appropriate a lot of times. So maybe we need more training to see, see the appropriateness in escalation.”*

(FGD007, Junior pharmacist)

## 4. Discussion

Our study provided deeper insights into the challenges faced by non-ASP hospital pharmacists in antimicrobial stewardship and identified facilitators and barriers to their greater involvement in antimicrobial stewardship in the three largest acute-care hospitals in Singapore. Similar to many developed countries worldwide, these hospitals have established ASP teams with dedicated ASP pharmacists for antimicrobial stewardship [24]. However, with the continued increase in antimicrobial use, the much larger pool of non-ASP hospital pharmacists remains an untapped resource for antimicrobial stewardship [25]. Despite the lack of formal recognition of their roles in antimicrobial stewardship, these pharmacists were observed to actively identify inappropriate antibiotic prescriptions when reviewing medication orders but were constrained by circumstances and self-efficacy to intervene regularly and confidently.

At the intrapersonal level, hospital pharmacists identified themselves as reviewers for drug safety before dispensing antibiotics, confining themselves to a restricted advisory role to the prescribing physician. This observation corroborated findings from other studies, which similarly identified that pharmacists were viewed as drug advisors rather than integral contributors to antibiotic decision-making, and their interventions on antibiotic prescribing often went unnoticed [26]. As such, recognition of the role that hospital pharmacists already play in antimicrobial stewardship and empowering them to play a formal role in joint decision-making in antibiotic prescribing can fully harness the untapped potential of hospital pharmacists in enhancing ASPs in hospitals [25].

Pharmacist-led rounds have been shown to improve patient care and also provide the mechanism for hospital pharmacists to be formally involved in antibiotic decisions. A study in Australia (evaluating a pharmacist-led penicillin allergy de-labelling ward round) reported a significant decrease in the use of restricted antibiotics and an increase in intravenous-to-oral conversion of antibiotics; the medical team’s compliance to the pharmacists’ interventions was as high as 81.5% [27]. A systematic review that evaluated pharmacist-led interventions in hospital settings further demonstrated improvements in the quality of medication use, reduction in hospital visits, and duration of hospital stay [28]. Pharmacist-led ward rounds have led to more judicious use of antibiotics in hospitals [29], but also promoted shared responsibility between pharmacists and physicians for appropriate antibiotic prescribing and provided a platform for pharmacists to have their interventions formally acknowledged, minimising pharmacists’ intervention fatigue and boosting their confidence [14,30].

The involvement of hospital pharmacists in antimicrobial stewardship was observed to be limited by knowledge deficiencies in clinical medicine and microbiology. Studies have suggested the use of advanced electronic medical records and mobile applications to help pharmacists keep up-to-date with hospital-specific antibiograms and guidelines, and the latest clinical knowledge [31,32,33]. Formal education and accreditation of non-ASP pharmacists in antimicrobial stewardship by hospital ASP teams, in addition to on-the-job training, could enhance their contributions to antimicrobial stewardship.

At the interpersonal level, despite frequent communications with physicians, pharmacists felt that the physicians did not trust their antibiotic recommendations. Strong rapport and collaboration between physicians and pharmacists are well recognised as important factors for ensuring the success of ASPs [10,34]. Through the interprofessional collaboration between pharmacists and physicians, hospital pharmacists’ expertise in pharmacology can be harnessed to optimise antibiotics for optimal clinical care, instead of confining hospital pharmacists to the role of antibiotic gatekeeping or policing [13,14]. ASPs in some institutions are moving away from the traditional physician-led antibiotic decision-making process and evolving towards a more collective approach involving general pharmacists and multidisciplinary staff, with an emphasis on shared responsibility for antibiotic prescribing [26]. Involving pharmacists in antibiotic decision-making would empower them in antimicrobial stewardship and also reduce medication errors and inappropriate antibiotic use, as well as improve patient care [35,36]. ASPs can be enhanced by empowering hospital pharmacists’ in their role as antibiotic stewards and facilitating strong pharmacist–physician collaborations in antibiotic prescribing decisions [37,38].

To ensure patients’ compliance with dispensed antibiotics, hospital pharmacists educate patients and caregivers on the purpose and appropriate use of antibiotics. In so doing, hospital pharmacists have subconsciously served as antibiotic stewards, increasing patients’ adherence to antibiotic use. A previous trial in Australia reported that involving community pharmacists in verbal patient education helped to improve antibiotic knowledge significantly and provided a personal touch for patients to understand the significance of antimicrobial resistance [39]. This could potentially be translated to the hospital setting where hospital pharmacists are engaged in patient education, helping to increase patients’ antibiotic knowledge and their awareness of antimicrobial resistance [30].

At the organisational level, hospital pharmacists found hospital-specific antibiotic guidelines to be useful but had mixed reactions to antibiotic CDSSs. That was despite the reported benefits of antibiotic CDSSs on optimising antibiotic utilisation and resulting in better clinical outcomes for patients [40,41]. However, this is not unexpected, as it was well known that antibiotic CDSSs were unable to provide comprehensive coverage on all aspects of antibiotic prescribing due to the complexity of antibiotic guidelines, posing major barriers to their implementation [42,43,44]. It is ideal that antibiotic recommendations can be individualised and dose optimised based on the unique pharmacokinetic profile of the patient (e.g., in the critically ill patient), and microbiological information (including susceptibility data) available. With the advancement of technology and data management systems, it is foreseeable that more sophisticated CDSSs will be developed to serve this very purpose in the near future [45]. Antibiotic CDSSs have been demonstrated to greatly improve the efficiency of antimicrobial stewardship interventions; similarly, the resource and time constraints that hospital pharmacists face in antimicrobial stewardship activities could be addressed by the same systems [46]. With the use of CDSSs, simple antimicrobial stewardship workflows can be incorporated, freeing up pharmacists’ time to review complex cases and participate in training and educational activities [47,48]. Therefore, to more effectively tap on the benefits of CDSS for antimicrobial stewardship, future studies could be embarked on to explore the facilitators and barriers to the acceptance and sustained use of CDSS by end users, including pharmacists.

In the community, hospital pharmacists can play an active role in public education on AMR and the appropriate use of antibiotics, as well as professional education of community/retail pharmacists on the local epidemiology and antibiotic susceptibility patterns. Some studies suggested the lack of comprehensive knowledge among community pharmacists on antimicrobial use and AMR [49]. Therefore, in order for community pharmacists to provide appropriate education and better engage with the public on antibiotic use, continuing professional education on antibiotics and AMR is crucial. Hospital pharmacists can be involved in a variety of educational platforms, including the development of educational leaflets and newsletters, speaking at conferences and public events, and providing on-the-job training [50,51].

Our study is limited to the perspective of hospital pharmacists in Singapore, a developed country with well-established ASPs in acute hospitals [6]. Nonetheless, the findings are applicable to other developed countries with established ASPs interested to enhance the ASPs by harnessing the untapped potential of hospital pharmacists in the hospitals. Furthermore, the strength of our study lies in the broad perspectives across institutions and levels of seniority, contributed by a large number of participants in FGDs. The grouping of participants by their seniority levels and the use of facilitators who were not clinically trained have also minimised social desirability bias and allowed for authentic responses from each FGD. Our previous work exploring the views of inpatient nurses on their roles in antimicrobial stewardship found that nurses played important roles as gatekeepers in the assurance of appropriate administration of prescribed antibiotics [52]. Future cross-disciplinary studies should be considered for triangulation of findings from the perspectives of physicians, nurses, and pharmacists, since one of the thrusts in Singapore’s National Strategic Action Plan on AMR is the optimisation of antimicrobial use through the further optimisation of antibiotic prescribing in hospitals [6].

## 5. Conclusions

Our findings reflected several significant themes on hospital pharmacists’ perspectives on their roles in antimicrobial stewardship. Barriers included low self-efficacy due to the perceived lack of clinical knowledge and experience, lack of recognition as joint antibiotic decision-makers, time constraints, and competing work demands. Facilitators included the natural inclination towards checking for medication safety and educating patients and caregivers on antibiotic use. Formalising the role of hospital pharmacists as antibiotic stewards and providing them with the required resources and training will capitalise on their untapped potential to enhance ASPs and further optimise antibiotic use in hospitals.

## Figures and Tables

**Table 1:** Participants’ characteristics.

Participants’ Characteristics		Number of Participants (*n* = 74)
Highest Education Level	Degree	55
	Masters	14
	PhD	5
Designation	Pharmacist	26
	Senior pharmacist	32
	Principal pharmacist (including senior clinical pharmacist/principal clinical pharmacist/senior principal clinical pharmacist/specialist pharmacist)	16
Gender	Male	16
	Female	58
Ethnic Group	Chinese	70
	Malay	1
	Indian	2
	Others	1
Years of practice in hospital	1 to 4 years	36
	5 to 9 years	26
	More than 10 years	12

## Data Availability

The data presented in this study are available on request from the corresponding author.

## Supplementary Materials

The following are available online at https://www.mdpi.com/article/10.3390/antibiotics10121441/s1, Supplementary File Annex S1: Consolidated criteria for reporting qualitative research (COREQ)—32 items (Tong et al., 2007) Supplementary File Annex S2: Interview Guide
