# Supplementary material for: Hospital Pharmacists and Antimicrobial Stewardship: A Qualitative Analysis
Source: Antibiotics (Basel). 2021 Nov 24;10(12):1441. doi: 10.3390/antibiotics10121441 (PMC8698014; doi:10.3390/antibiotics10121441)
Supplement: Supplementary file 1 [file antibiotics-10-01441-s001.zip › Proof_Antibiotics_SuppFileAnnex1_COREQ_final.pdf]

Supplementary File Annex 1: Consolidated criteria for reporting qualitative research (COREQ) – 32 items (Tong *et al* 2007)

No	Item	Guide questions / Descriptions	Response	Section reported under
**Domain 1: Research team and reflexivity**				
Personal characteristics				
1.	Interviewer/ Facilitator	Which author/s conducted the interview or focus group?	A total of 3 Research Assistants from the broader study team conducted the focus group discussions. They are not authors of this paper.	Materials and methods – *Data collection*
2.	Credentials	What were the researcher's credentials? E.g. PhD, MD	Researchers who conducted the focus group discussions were minimally with a Bachelor's degree.	Materials and methods – *Data collection*
3.	Occupation	What was their occupation at the time of the study?	All researchers were Research Assistants at the time of the study.	Materials and methods – *Data collection*
4.	Gender	Was the researcher male or female?	All researchers were females.	Materials and methods – *Data collection*
5.	Experience and training	What experience or training did the researcher have?	All researchers were trained in qualitative data collection techniques.	Materials and methods – *Data collection*
Relationship with participants				
6.	Relationship established	Was a relationship established prior to study commencement?	Relationship was established at the point of informed consent taking before each focus group discussion.	N/A
7.	Participant knowledge of the interviewer	What did the participants know about the researcher? e.g. personal goals, reasons for doing the research	The names, occupations, and reasons for carrying out the research were made known to the participants.	N/A
8.	Interviewer characteristics	What characteristics were reported about the interviewer/facilitator? e.g. Bias, assumptions,	The interviewer/facilitator did not have any personal bias, assumptions, reasons and interests in the research topic.	N/A

		reasons and interests in the research topic		
**Domain 2: Research team and reflexivity**				
Theoretical framework				
9.	Methodological orientation and Theory	What methodological orientation was stated to underpin the study? e.g. grounded theory, discourse analysis, ethnography, phenomenology, content analysis	Thematic analysis was performed.	Materials and methods – *Data analysis*
Participant selection				
10.	Sampling	How were participants selected? e.g. purposive, convenience, consecutive, snowball	Participants were purposively sampled and grouped according to their seniority (i.e. Junior pharmacist, Senior pharmacist, Principal/Clinical/Specialist pharmacist).	Materials and methods – *Study design and study population*
11.	Method of approach	How were participants approached? e.g. face-to-face, telephone, mail, email	Invitation letters were sent out through the pharmacy department in each hospital. Interested pharmacists would contact the study team and they would be purposively selected.	Materials and methods – *Study design and study population*
12.	Sample size	How many participants were in the study?	74 pharmacists were recruited into the study.	Results
13.	Non-participation	How many people refused to participate or dropped out? Reasons?	None. Participants who were not interested to take part in the study did not contact the study team.	N/A
Setting				
14.	Setting of data collection	Where was the data collected? e.g. home, clinic, workplace	The FGDs were conducted in a closed-door meeting room within the respective hospital premises.	Materials and methods – *Data collection*
15.	Presence of non-participants	Was anyone else present besides the participants and researchers?	No. Researchers from the study team and participants were the only ones present.	N/A
16.	Description of sample	What are the important characteristics of the sample? e.g.	Demographic data collected were tabulated in Table 1.	Results

		demographic data, date		
Data collection				
17.	Interview guide	Were questions, prompts, guides provided by the authors? Was it pilot tested?	Interview guide was provided as Supplementary File (Annex 2) and it was pilot tested prior to use.	Materials and methods – *Data collection*
18.	Repeat interviews	Were repeat interviews carried out? If yes, how many?	No, repeat interviews were not carried out.	N/A
19.	Audio/visual recording	Did the research use audio or visual recording to collect the data?	Audio recording was used to collect the data.	Materials and methods – *Data collection*
20.	Field notes	Were field notes made during and/or after the interview or focus group?	Yes, field notes were made during the focus group discussions.	N/A
21.	Duration	What was the duration of the interviews or focus group?	Each focus group discussion lasted between 1.5-2hours.	Materials and methods – *Data collection*
22.	Data saturation	Was data saturation discussed?	Yes, data saturation was discussed.	Materials and methods – *Data analysis*
23.	Transcripts returned	Were transcripts returned to participants for comment and/or correction?	No, transcripts were not returned to the participants for comments and/or corrections.	N/A
**Domain 3: Analysis and findings**				
Data analysis				
24.	Number of data coders	How many data coders coded the data?	Three coders coded the data.	Materials and methods – *Data analysis*
25.	Description of the coding tree	Did authors provide a description of the coding tree?	Coding tree was not provided.	N/A
26.	Derivation of themes	Were themes identified in advance	Themes were derived from the data.	Materials and methods –

		or derived from the data?		*Data analysis*
27.	Software	What software, if applicable, was used to manage the data?	QSR International's NVivo 12 software was used to manage the data.	Materials and methods – *Data analysis*
28.	Participant checking	Did participants provide feedback on the findings?	No, participants did not provide feedback on the findings.	N/A
Reporting				
29.	Quotations presented	Were participant quotations presented to illustrate the themes / findings? Was each quotation identified? e.g. participant number	Yes, participant quotations were presented and each quotation was identified.	Results
30.	Data and findings consistent	Was there consistency between the data presented and the findings?	Yes, there were consistency between data presented and the findings.	Results and Discussion
31.	Clarity of major themes	Were major themes clearly presented in the findings?	Yes, major themes were clearly presented in the findings.	Results
32.	Clarity of minor themes	Is there a description of diverse cases or discussion of minor themes?	Yes, minor themes were discussed.	Results and Discussion
