# Supplementary material for: Hospital Pharmacists and Antimicrobial Stewardship: A Qualitative Analysis
Source: Antibiotics (Basel). 2021 Nov 24;10(12):1441. doi: 10.3390/antibiotics10121441 (PMC8698014; doi:10.3390/antibiotics10121441)
Supplement: Supplementary file 1 [file antibiotics-10-01441-s001.zip › Proof_Antibiotics_SuppFileAnnex2_InterviewGuide_final.pdf]

Supplementary File Annex 2: Interview Guide (Focus Group Discussion)

Determinants of antibiotic use and perceptions of antibiotic resistance among inpatient pharmacists in Singapore hospitals.

1. **Clinical, psychosocial, organisational, and cultural determinants for antibiotic prescribing among prescribers; Interaction between prescribers and non-prescribers**

- 1) What types of ward do you work in, 2) the number of years you have worked in this hospital, and 3) situations when you will manage antibiotics in the hospital.
- What do you think about the current antibiotic use in your institution?
- In the hospital, care is usually delivered in a team. Please describe your interaction with the patient's care team (which includes physicians, nurses, their caregivers, and perhaps your fellow pharmacists) and how this interaction affects your decision to verify/approve an antibiotic order.
- Please describe situations when you would consider disagreeing with a physician's order and not verify/approve an antibiotic order.
- Please give an example when you are personally unsure of the 1) choice/spectrum (escalate/de-escalate) and 2) dosage of antibiotics and duration (discontinue).
- How comfortable will you be to seek advice from your seniors, peers, or the rest of the care team (including physicians and nurses) when in doubt?

2. **Guidelines for antibiotics management in the hospital**

- Please share with me some specific protocols or guidance associated with antibiotic prescribing and management in your hospital?
- Would you mind sharing where these guidelines or protocols are available in your institution?
- Please describe what were your experiences in using [above answer] to guide your work with regards to antibiotic prescribing (for physicians and nurses) and verification/review (for pharmacists)?
- How was your experience using it? What are some of the common feedback on the ground?

3. **Awareness, perception, and attitudes towards antibiotic resistance**

- On a personal level, how do you think the antibiotic resistance issue affects you?
- What are some of the contributing factors for antibiotic resistance?
- How is antibiotic resistance affecting the way care is delivered in the hospitals?

- How do you think the hospital sector can contribute to preventing the progression of antibiotic resistance?

- Would you mind describing what are some facilitators affecting the successful management of antibiotic resistance in hospitals?

- What are some barriers affecting the successful management of antibiotic resistance in hospitals?

- To what extent do you feel empowered or not empowered in playing a role in managing the antibiotic resistance issue in Singapore?

4. Suggestions on ways to improve awareness of antibiotic use and antibiotic resistance

- How would you describe the current knowledge healthcare workers have about antibiotic use and antibiotic resistance?

- What about their perceptions and attitudes towards the same issue?
- What do you think are some of the gaps that can be explored to further improve antibiotic use?

- Are there any other suggestions?

The End
